# Changes in the numbers of patients with acute gastroenteritis after voluntary introduction of the rotavirus vaccine in a Japanese children’s primary emergency medical center

**DOI:** 10.1186/s12199-017-0638-3

**Published:** 2017-03-31

**Authors:** Ichiro Morioka, Naohiro Kamiyoshi, Masahiro Nishiyama, Tomohiko Yamamura, Shogo Minamikawa, Sota Iwatani, Hiroaki Nagase, Kandai Nozu, Noriyuki Nishimura, Mariko Taniguchi-Ikeda, Kazuto Ishibashi, Akihito Ishida, Kazumoto Iijima

**Affiliations:** 1 Department of Pediatrics, Kobe University Graduate School of Medicine, 7-5-2, Kusunoki-cho, Chuo-ku, Kobe, 650-0017 Japan; 2 Kobe Children’s Primary Emergency Medical Center, Kobe, 6510073 Japan

**Keywords:** Children, Gastroenteritis, Norovirus, Rotavirus, Vaccination

## Abstract

**Background:**

Acute gastroenteritis (AGE) is a major reason for presentation to pediatric primary emergency medical centers. Because rotavirus vaccines were introduced in November 2011 for voluntary vaccination in Japan, we analyzed the changes in the numbers of AGE patients.

**Methods:**

The number and proportion of patients visiting Kobe children’s primary emergency medical center from January 2011 to February 2015 due to AGE, out of all visiting children, were investigated retrospectively. The rotavirus and norovirus epidemic periods were defined as the periods from March to June and from November to February, respectively, based on their disease prevalence.

**Results:**

In patients ≤2 years of age, the numbers and proportions of patients with AGE were significantly decreased from 2464/14098 (17%) in 2011 to 1888/12321 (15%) in 2014 (*p* < 0.01). In patients ≤2 and 3–5 years of age, significant decreases in AGE patients between 2011 and 2014 were observed during the rotavirus season (from 20% [1090/5329] to 14% [642/4482] in patients aged ≤2 years and from 23% [704/3047] to 20% [572/2807] in patients aged 3–5 years, *p* < 0.01 and *p* < 0.05, respectively), but not during the norovirus season (from 19% [834/4436] to 19% [797/4160] in patients aged ≤2 years and from 20% [679/3334] to 25% [710/2852] in patients aged 3–5 years).

**Conclusions:**

The estimated rotavirus vaccine coverage in our area increased from 1% in 2011 to 49% in 2014; this coverage may have resulted in a reduction in AGE patients, both directly and indirectly, in our Japanese children’s primary emergency medical center.

## Background

Worldwide, rotavirus is a common causative virus of acute gastroenteritis (AGE) in children [1], and, in 2008, approximately 453,000 rotavirus AGE-related deaths occurred in children younger than 5 years of age [2]. Although death is relatively rare in Japan, AGE due to rotavirus represents a heavy disease burden for providing medical treatment not only in hospitals, but also in pediatric outpatient clinics [3–5]. In Japan, approximately 26,000–78,000 hospitalizations and 800,000 visits to outpatient clinics or outpatient department of hospitals due to rotavirus AGE have been estimated annually in children under school age [3–5]. From many countries where the rotavirus vaccine has already been introduced, the direct effects thereof have been reported, including reduced numbers of pediatric AGE patients requiring hospitalization, outpatient visits, and emergency department visits [6–14].

A live attenuated monovalent human rotavirus vaccine (Rotarix™: GlaxoSmithKline Biologicals, Rixensart, Belgium) and a pentavalent human-bovine reassortant rotavirus vaccine (RotaTeq™: Merck & Co., Inc., New Jerzy, USA) were introduced for voluntary vaccination in Japan in November 2011 and July 2012, respectively. Rotarix™ is derived from the single-strain human rotavirus with G1P[8] genotype and RotaTeq™ includes 5 bovine-human recombinant rotaviruses with genotypes of G1P[5], G2P[5], G3P[5], G4P[5], and G6P[8]. An early study showed reduced rates of severe pediatric AGE due to rotavirus after the introduction of voluntary rotavirus vaccination at 3 pediatric outpatient clinics in Shibata-city, Niigata prefecture, Japan [15, 16]. However, the direct and indirect effects of this vaccine have not yet been studied in a children’s primary emergency medical center, which is a unique Japanese medical service system.

Kobe children’s primary emergency medical center provides medical services for pediatric patients without trauma, only during holidays and outside of regular working hours. Approximately 30,000 pediatric patients per year visit this center, which covers 50–60% of all primary emergency patients in Kobe-city (52% in 2011, 57% in 2012, 59% in 2013, and 58% in 2014). More than 130 pediatricians are working on a part-time basis.

The purpose of this study was to analyze the changes in the numbers and proportions of patients with AGE and severe AGE after the voluntary-based introduction of rotavirus vaccines in Kobe children’s primary emergency medical center. Moreover, we also investigated if there were any changes in the numbers and proportions of AGE and severe AGE patients specifically during the rotavirus and norovirus epidemic seasons.

## Methods

### Subjects and analysis

All patients ≤ 15 years of age who visited Kobe children’s primary emergency medical center from January 1, 2011 to February 28, 2015 were enrolled in this retrospective study. No exclusion criteria were established. Patients were classified into three subgroups based on age; ≤ 2, 3–5, and ≥ 6 years of age. The AGE patients were identified from the medical database based on the diagnosis of AGE, and the numbers of AGE patients in the full season, rotavirus season, and norovirus season were calculated from 2011 to 2014. The proportions of AGE and severe AGE patients in each season were calculated based on the number of total patients who visited our center. In patients ≤ 2 years of age, the numbers and proportions for each season in 2012, 2013, and 2014 were compared with those in 2011, when the rotavirus vaccines were just introduced in Japan, which served as the baseline. Patients with 3 to 5 years of age, or ≥ 6 years of age were also analyzed for investigating indirect effects of vaccination. In the examined stools of AGE patients, the detection numbers and rates of rotavirus during the rotavirus season from 2011 to 2014 were analyzed. Finally, from the medical database, the number of patients < 12 months of age with a confirmed diagnosis of intussusception were calculated for each year, because intussusception is a major side effect of the rotavirus vaccine [17–19].

The study design and data collection for this study were approved by the Ethical Committee of Kobe Children’s Primary Emergency Medical Center (no. 57-4; approved on November 7, 2014). All procedures performed in studies involving human participants were in accordance with the ethical standards of the institutional and national research committee and with the 1964 Helsinki declaration and its later amendments or comparable ethical standards. For this type of study formal consent is not required.

### Definitions

The diagnosis of AGE was made at the discretion of the attending pediatrician based on the clinical manifestations, such as vomiting, diarrhea, fever, and abdominal pain, and only some patients were confirmed to have the causative virus in their stool using an antigen detection kit. AGE patients who required intravenous infusion and/or hospitalization were defined as “severe”. “Full season” was defined as the period between January and December each year. The two major causative viruses for AGE are rotavirus and norovirus [13, 20]. Currently, a norovirus vaccine is not commercially available. The “rotavirus season” was defined as the rotavirus epidemic period between March and June, and the “norovirus season” as the norovirus epidemic period between November and February, based on the reported prevalence in Japan [21].

### Estimated vaccine coverage in Hyogo prefecture

The estimated vaccine coverage in Hyogo prefecture was calculated using the following formula: (estimated vaccine coverage, %) = 100 × [(shipping number of Rotarix™/2 in a year) + (shipping number of RotaTeq™/3 in a year)]/(the birth number in a year), because Rotarix™ is inoculated 2 times before 6 months of age and RotaTeq™ is inoculated 3 times before 8 months of age for infants. The data of the shipping number in Hyogo prefecture were obtained from the pharmaceutical companies of the rotavirus vaccines.

### Statistical analysis

Data are presented as the number (percentage). Chi-square tests were performed using Excel Statistics (Statcel 3; Social Survey Research Information Co. Ltd., Tokyo, Japan). For all analyses, *p* < 0.05 was considered statistically significant.

## Results

### Total and AGE patients during the study period

**Fig. 1:**
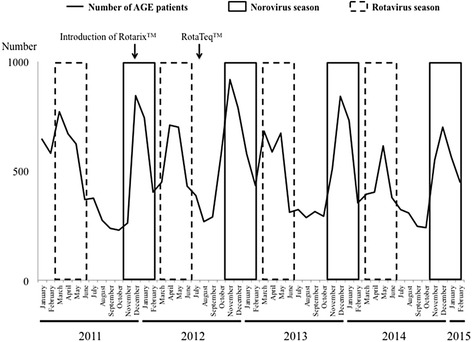
Monthly numbers of AGE patients ≤ 15 years of age from January 1, 2011 to February 28, 2015. *Black squares* indicate the norovirus epidemic seasons. *Dotted squares* indicate rotavirus epidemic seasons. AGE, acute gastroenteritis

A total of 125,968 patients ≤ 15 years of age visited Kobe children’s primary emergency medical center from January 1, 2011 to February 28, 2015. The total numbers of patients ≤ 2, 3–5, and ≥ 6 years of age were 56,641, 34,419, and 34,908, respectively. The total number of AGE patients was 23,615 and the proportion of AGE patients of the total number of patients was 18.7%. The monthly trends of AGE patients in our center from January 2011 to February 2015 are shown in Fig. 1. For each year, the epidemic of AGE showed a binomial distribution in spring and winter.

### Patients ≤ 2 years of age

**Fig. 2:**
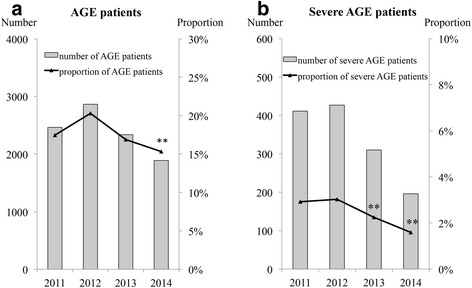
Numbers and proportions of **a** AGE and **b** severe AGE patients ≤ 2 years of age. *Gray columns* indicate the numbers of AGE or severe AGE patients ≤ 2 years of age in the full season each year. The *line* indicates the proportions of AGE or severe AGE patients based on the total number of visiting patients. ** *p* < 0.01 compared with in 2011. AGE, acute gastroenteritis

In the full season, a total of 14,098, 14,112, 13,826, and 12,321 patients ≤ 2 years of age visited the center in 2011, 2012, 2013, and 2014, respectively. The numbers and proportions of AGE and severe AGE patients ≤ 2 years of age in the full season each year are shown in Fig. 2. The proportion of AGE patients was significantly decreased from 17.4% in 2011 to 15.3% in 2014 (*p* < 0.01), but not in 2012 (20.3%) or 2013 (16.9%). The proportion of severe AGE patients was significantly decreased from 2.9% in 2011 to 2.2% in 2013 and 1.6% in 2014 (*p* < 0.01), whereas no difference was seen in 2012 (3.0%).

**Fig. 3:**
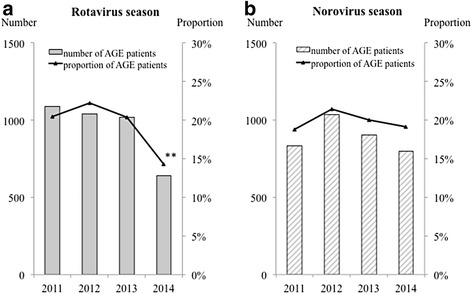
Numbers and proportions of AGE patients ≤ 2 years of age in the **a** rotavirus and **b** norovirus epidemic seasons. *Gray columns* and *gray stripe columns* indicate the numbers of AGE patients ≤ 2 years of age in the rotavirus season and norovirus season each year, respectively. The *line* indicates the proportions of AGE patients based on the total numbers of visiting patients. ** *p* < 0.01 compared with in 2011. AGE, acute gastroenteritis

**Fig. 4:**
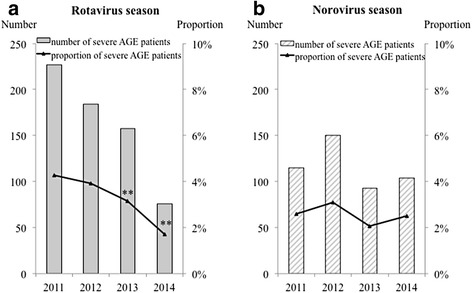
Numbers and proportions of severe AGE patients ≤ 2 years of age in the **a** rotavirus and **b** norovirus epidemic seasons. *Gray columns* and *gray stripe columns* indicate number of severe AGE patients ≤ 2 years of age in the rotavirus season and norovirus season each year, respectively. The *line* indicates the proportions of severe AGE patients based on the total number of visiting patients. ** *p* < 0.01 compared with in 2011. AGE, acute gastroenteritis

The annual trends of the numbers and proportions of AGE and severe AGE patients in the rotavirus season and norovirus season are shown in Figs. 3 and 4. The proportion of AGE patients was significantly decreased from 20.4% in 2011 to 14.3% in 2014 in the rotavirus season (*p* < 0.01). However, no significant decrease was found from 2011 to 2014 in the norovirus season. The proportion of severe AGE patients was also significantly decreased from 4.3% in 2011 to 3.2% in 2013 and 1.7% in 2014 in the rotavirus season (*p* < 0.01), whereas there was no significant decrease in the norovirus season.

### Patients 3–5 years of age

The numbers and proportions of AGE and severe AGE patients aged 3–5 years are shown in Table 1. The proportion of severe AGE patients was significantly decreased from 3.9% in 2011 to 3.2% in 2014 (*p* < 0.05), however, no decrease was found in the proportions of AGE patients (19.8% in 2011 and 21.1% in 2014). In the rotavirus season, the proportions of AGE and severe AGE patients were significantly decreased from 23.1% and 5.2% in 2011 to 20.4% and 3.2% in 2014 (*p* < 0.05 and *p* < 0.01, respectively). On the other hand, in the norovirus season, no significant decrease in the proportions of AGE and severe AGE patients was found between 2011 and 2014.

**Table 1:** Numbers and proportions of AGE and severe AGE patients aged 3 to 5 years

	Full season				Rotavirus season				Norovirus season			
	2011	2012	2013	2014	2011	2012	2013	2014	2011	2012	2013	2014
Total number of patients	8484	8510	7992	7781	3047	2771	2625	2807	3334	3089	3093	2852
AGE patients	1681 (20)	1974 (23)	1735 (22)	1647 (21)	704 (23)	696 (25)	643 (24)	572 (20)^*^	679 (20)	782 (25)	760 (25)	710 (25)
Severe AGE patients	331 (4)	343 (4)	293 (4)	247 (3)^*^	159 (5)	135 (5)	128 (5)	89 (3)^**^	129 (4)	121 (4)	114 (4)	124 (4)

Data are presented as n (%). AGE, acute gastroenteritis

^*^ *p* < 0.05 and ^**^ *p* < 0.01 compared with in 2011

### Patients ≥ 6 years of age

The numbers and proportions of AGE and severe AGE patients ≥ 6 years of age are shown in Table 2. No change was observed in the proportion of AGE patients between 2011 and 2014. A significant decrease in the rotavirus season was found between 2011 and 2012 but not between 2011 and 2014.

**Table 2:** Numbers and proportions of AGE and severe AGE patients aged ≥ 6 years

	Full season				Rotavirus season				Norovirus season			
	2011	2012	2013	2014	2011	2012	2013	2014	2011	2012	2013	2014
Total number of patients	8306	8669	7522	7941	2775	2816	2399	2591	4036	3333	3527	3765
AGE patients	1730 (21)	1830 (21)	1758 (23)	1706 (21)	634 (23)	552 (20)^**^	588 (25)	577 (22)	740 (18)	900 (27)	771 (22)	753 (20)
Severe AGE patients	407 (5)	385 (4)	329 (4)	332 (4)^*^	161 (6)	112 (4)^**^	132 (6)	123 (5)	169 (4)	190 (6)	147 (4)	165 (4)

Data are presented as n (%). AGE, acute gastroenteritis

^*^ *p* < 0.05 and ^**^ *p* < 0.01 compared with in 2011

### Detection number and rate of rotavirus

Detection numbers and rates of rotavirus in AGE patients during the rotavirus season from 2011 to 2014 were shown in Table 3. In the examined stools of AGE patients, the detection rate was significantly decreased from 67% in 2011 to 27% in 2014 (*p* < 0.01).

**Table 3:** Detection numbers and rates of rotavirus in AGE patients during the rotavirus season

	2011	2012	2013	2014
Examined stools of AGE patients, n	84	96	79	30
Positive result of rotavirus, n	56	55	47	8
Detection rate, %	66.7	57.3	59.5	26.7^**^

AGE, acute gastroenteritis

^**^ *p* < 0.01 compared with in 2011

### Number of intussusceptions

The numbers of patients < 12 months of age with a confirmed diagnosis of intussusception were 2, 3, 3, and 4 in 2011, 2012, 2013, and 2014, respectively, without a statistical difference during the study period (*p* = 0.79).

### Estimated vaccine coverage in Hyogo prefecture

The estimated vaccine coverage rates in Hyogo prefecture were 1% in 2011, 25% in 2012, 42% in 2013, and 49% in 2014.

## Discussion

In Japan, the rotavirus vaccine was first introduced in November 2011 on a voluntary basis. Herein, we analyzed the changes in the numbers and proportions of patients with AGE and severe AGE after the voluntary-based introduction of rotavirus vaccines in Kobe children’s primary emergency medical center. As results, in patients ≤ 2 and 3–5 years of age, the proportions of AGE and severe AGE patients based on the total number of patients who visited our center were significantly decreased between 2011 and 2014 in the rotavirus season, but not in the norovirus season. In patients ≥ 6 years of age, the years in which we observed a significant decrease of the proportions of severe AGE patients compared to in 2011 did not correspond between the full season and rotavirus season. The rotavirus vaccine coverage in our area increased to 49% in 2014. This coverage may have resulted in a reduction in the numbers of patients ≤ 2 and 3–5 years of age presenting with AGE and severe AGE in the rotavirus season in our Japanese primary emergency medical center.

Rotavirus vaccine is inoculated to infants < 6 months of age for Rotarix™ or < 8 months of age for RotaTeq™. Therefore, the reductions in AGE and severe AGE patients ≤ 2 years of age in the rotavirus season might represent direct effects of vaccination. In patients ≤ 2 years of age, severe AGE and AGE were significantly decreased in 2013 and 2014, and 2014, respectively, as compared in 2011. Oishi et al. reported a significant reduction of severe rotavirus AGE 1 year after the vaccine introduction (estimated vaccine coverage, 33%) [15].

Another reason of the reduction of AGE patients may be the increase in preventive measures such as improved hand hygiene and environmental disinfection during the study period. And the reduction of severe AGE patients may associate with the increase in use of oral rehydration solution at home. However, because there was no reduction in the proportion of AGE and severe AGE patients during the norovirus season, these were likely not major reasons for the reduction of AGE and severe AGE patients.

Because our study calculated the proportion of AGE or severe AGE patients based on the total number of visiting patients, the data may be influenced by epidemics of other viruses such as influenza. The epidemic season of influenza is usually the same as that of norovirus in Japan. Although, during the study period, the highest number of influenza patients visited our center in 2012 (data not shown), no decrease in the percentage of AGE or severe AGE was found in 2012 during the full and norovirus seasons, indicating that there was likely no major influence of influenza on the results.

Some previous studies have shown decreased rates of AGE and severe AGE after the rotavirus vaccine introduction for infants in adults and older children as well (indirect effects of vaccine) [14, 22, 23]. In the present study, patients older than 3 years of age during the study period were not administered the rotavirus vaccine (except for a small number of patients aged 3 years in 2014 who were inoculated with Rotarix™ in 2011). Our results thus suggest that indirect effects of the rotavirus vaccine appeared in children aged 3–5 years, but not in children ≥ 6 years of age. Although the detailed reasons of this discrepancy are not clear, the vaccine coverage might still be too low to show indirect effects for children ≥ 6 years of age.

Finally, intussusception is a major side effect of the rotavirus vaccine, especially within 7 days after the first inoculation [18, 19]. Therefore, we investigated the number of cases of intussusception in infant patients. In our center, there was no increase in the number of patients with intussusception over time, even as the vaccine coverage increased.

This study has some limitations: 1) the causative virus was not always determined from stool samples of the AGE patients, because this study was conducted in a primary emergency medical center where we provide only primary medical care or screening of patients with severe conditions requiring hospitalization. We cannot accurately determine if the reduction of AGE patients was due to the reduction of rotavirus AGE patients. Although the limited AGE patients were examined and the number of examined stools varied each year, the detection rate of rotavirus in 2014 significantly decreased as compared with that in 2011. The trends of AGE patients caused by rotavirus in our center corresponded with the national epidemiological data [21]. To make up for this major limitation, we classified the study period into 2 seasons based on the prevalence of rotavirus infections in Japan [21]: the “rotavirus season” and “norovirus season,” and clearly showed the reduction of AGE patients only in the rotavirus season. 2) The criteria of severe AGE were not uniform, owing to the fact that the study was conducted at a large primary emergency medical center where more than 130 pediatricians are working on a part-time basis. 3) The rotavirus vaccine inoculation rate in patients who developed AGE and severe AGE was not known. If we can obtain this data, the vaccine effects may be assessed accurately. 4) This study used the patient number in 2011 as a reference. The ideal reference period should be included the years before the rotavirus vaccine introduction, such as 2009 to 2011. However, because our children’s primary emergency medical center was opened in December, 2010, we could not expand the reference period. Despite these limitations, this study, for the first time, clearly showed the changes in the numbers and proportions of patients with AGE after the voluntary introduction of the rotavirus vaccine in a Japanese children’s primary emergency medical center.

## Conclusions

The numbers and proportions of AGE and severe AGE patients not only aged ≤ 2 years, but also 3–5 years, decreased in the rotavirus season 3 years after the voluntary introduction of the rotavirus vaccine in a Japanese children’s primary emergency medical center. Although the epidemic trends of AGE vary each year, the rotavirus vaccine may lead to a decreased AGE burden for infants and young children.

## Abbreviation

AGE: Acute gastroenteritis
